# Occupational physicians dealing with mental health: between employee and company interests: a qualitative study

**DOI:** 10.1186/s40359-022-01012-2

**Published:** 2022-12-14

**Authors:** Tim Pößnecker, Maximilian Baxendale, Simone Braun, Elena Schwarz, Michael Hölzer, Peter Angerer, Harald Gündel, Elisabeth Balint, Eva Rothermund

**Affiliations:** 1 Department of Psychosomatic Medicine and Psychotherapy, Ulm University Medical Center, Ulm, Germany; 2 Leadership Personality Center Ulm (LPCU), Ulm, Germany; 3 Institute of Occupational and Social Medicine, Düsseldorf University Medical Center, Düsseldorf, Germany; 4 Sonnenbergklinik, ZfP Südwürttemberg, Stuttgart, Germany

**Keywords:** Occupational health physician, Mental health, Organizational culture, Common mental disorders, Qualitative research

## Abstract

**Background:**

Occupational health physicians are increasingly confronted with mental health issues at their workplace. Facing them, most of them feel insecure and not sufficiently trained. Employee’s mental well-being depends at the same time on individual and significantly on organizational variables. This complicates the physician’s position, since they have to serve many interests. The focus of the present study is to investigate what difficulties occupational health physicians face and how organizational culture and management influence their work.

**Methods:**

Interviews were conducted with N = 25 physicians as part of a training for basic mental health care. Interviews were interpreted using qualitative content analysis.

**Results:**

Working with mentally ill employees was difficult for the physicians interviewed. Many felt insecure managing and preventing mental health issues. A need for further education was observed. Environmental factors (organizational culture, management) have a strong impact on the work of an occupational health physician and highlight its systemic dimension. Even though many of our participants report a meanwhile more open attitude towards mental disorders at their workplace, on the level of direct contact to the management prevail descriptions of little acceptance and a high priority of economic outcomes.

**Conclusions:**

More education on topics of mental health is needed for occupational health physicians. Future trainings should consider the intertwined nature of their work and enable them in dealing consciously with other actors in the company. For enhancing employee’s mental well-being occupational health physicians could be granted a strengthened position in companies or be supported through more exchange with colleagues in other companies.

## Background

“*An emergency doctor saves lives. An occupational physician saves existences—But only if the company wants that*﻿” (Statement of an occupational health physician—I19).

Over the last years, issues of mental health at the workplace receive increasing attention. According to the WHO Global Burdens of Disease, mental disorders are a growing source of diminished quality of life [1] and account for a rising number of sick leave days [2]. Especially job characteristics such as high time pressure, frequent interruptions, high job insecurity and conflicts at work have a negative impact on mental health [3]. Mental disorders are observed globally in high- as well as low-income countries [4–6]. Even in high-income countries with a high standard health system people most in need do not receive adequate treatment [6]. Common mental illnesses such as depression and anxiety-disorders are also growing risk factors for well-being at the workplace and to the ability to work and perform at high requirements. An important influence on mental disorders are unfavorable working conditions [7–10], especially for already mentally ill employees [11].

Furthermore, mental health topics are recently receiving more attention in many companies. Concepts such as psychosocial safety climate increasingly influence the attitude of companies towards mental health issues, put mental well-being of employees on top of the company’s agenda and have a proven impact on the mental health of employees [12, 13]. But at the same time there remains insecurity about how to tackle these problems. Companies struggle to implement evidence-based programs which can prevent or reduce mental health problems at work [14–19].

The degree to which issues of mental health are considered important in a company is, apart from individual factors, dependent on a series of variables. One of these variables is the organizational culture of a company. According to Schein’s model of organizational culture, culture is defined on three different levels: 1) artifacts and behavior, 2) espoused values and 3) assumptions [20]. The research on organizational culture [21] has only scarcely focused on the impact on mental health in a company, but nevertheless shown that favorable organizational culture leads to lower work-related stress among employees [22–24].

Mental health of employees is highly affected by their managers leadership style [25, 26]. Leadership style affects employee’s health in general as well as explicitly regarding job satisfaction, job well-being, sickness absences and job performance [27]. Behavior of managers, e.g., in the form of problem-solving skills, work-planning ability and participating leadership style has a strong predictive power on all kinds of health outcomes [28].

This is of special importance for occupational health physicians (= OHP), since they have a central, often conflicting or even ambiguous role [29] in companies: they deal with employees as patients and are familiar with company structures and interests. Therefore, their role in respect to their employees varies between a sometimes therapeutic relationship (as in other medical professions) with a strong focus on prevention and medical care and in other situations a more detached relationship (for example when doing assessments).

Because of the developments mentioned above OHPs are faced with issues such as a growing number of employees with psychological distress or even mental disorders seeking help [30] and a changing societal attitude towards mental health issues. Concepts as the research on safety climates [31, 32] and more specific psychosocial safety climate [33, 34] highlight this grown importance of employee safety, well-being and their significance for mental health [35] and are therefore a support for OHPs, but also a challenge.

Many OHPs feel not sufficiently prepared for dealing with these challenges [36] and are furthermore insecure, when cooperating or communicating with other providers of the health system [37–39]. Often, they struggle how to handle the conflicting ethical demands of their position [40]. In countries such as the Netherlands, these insecurities have led to the implementation of structured guidelines to assist OHPs in dealing with mentally ill employees [41, 42]. In Germany however, no such guidelines exist. Therefore, an insecurity often remains how to tackle mental illnesses at the workplace. New concepts such as the psychosomatic consultation at the workplace [43, 44] offer employees the possibility to see a specialized psychotherapist directly in their workplace are not yet widely implemented. Other interventions such as modified Balint-Groups (= groups of clinicians, discussing about interactions with difficult clients under supervision) adapted to workplace related topics [45] also provide support in the form of group sessions for employees and managers, but are also only available for few persons. Thus, an increasing number of OHPs decides to undergo a voluntary, comprehensive theoretical and clinical training course in basic mental health care (German: “Psychosomatische Grundversorgung für Arbeits- und Betriebsmediziner”).

There is rather scarce empirical evidence that dealing with mental illness is an especially challenging task for OHPs [46]. The question which organizational factors influence the management of mental ill-health by an OHP in which way and how this complicates the work of an OHP is not yet sufficiently answered. Since OHPs attending a training on basic mental health care should have expert insight in mental health care problems for an OHP, we chose to interview them.

We were interested in the following research questions: How did OHPs perceive 1) their role in the management of mentally ill employees in their company, 2) the influence of the organizational culture and 3) the role of managers for employee’s mental health. Concerning these questions, we were also interested in how the OHPs attitude changed through visiting the training.

Since there already is a reliable body of literature on the interplay of management, organizational culture and mental health, we were primarily interested in assessing how OHPs felt about these issues and which—for example—insecurities or conflicts were experienced by them. This paper tries to answer these questions through the expert-view of OHPs with interest in basic mental health care.

## Methods

### Recruitment and observation

**Fig. 1:**
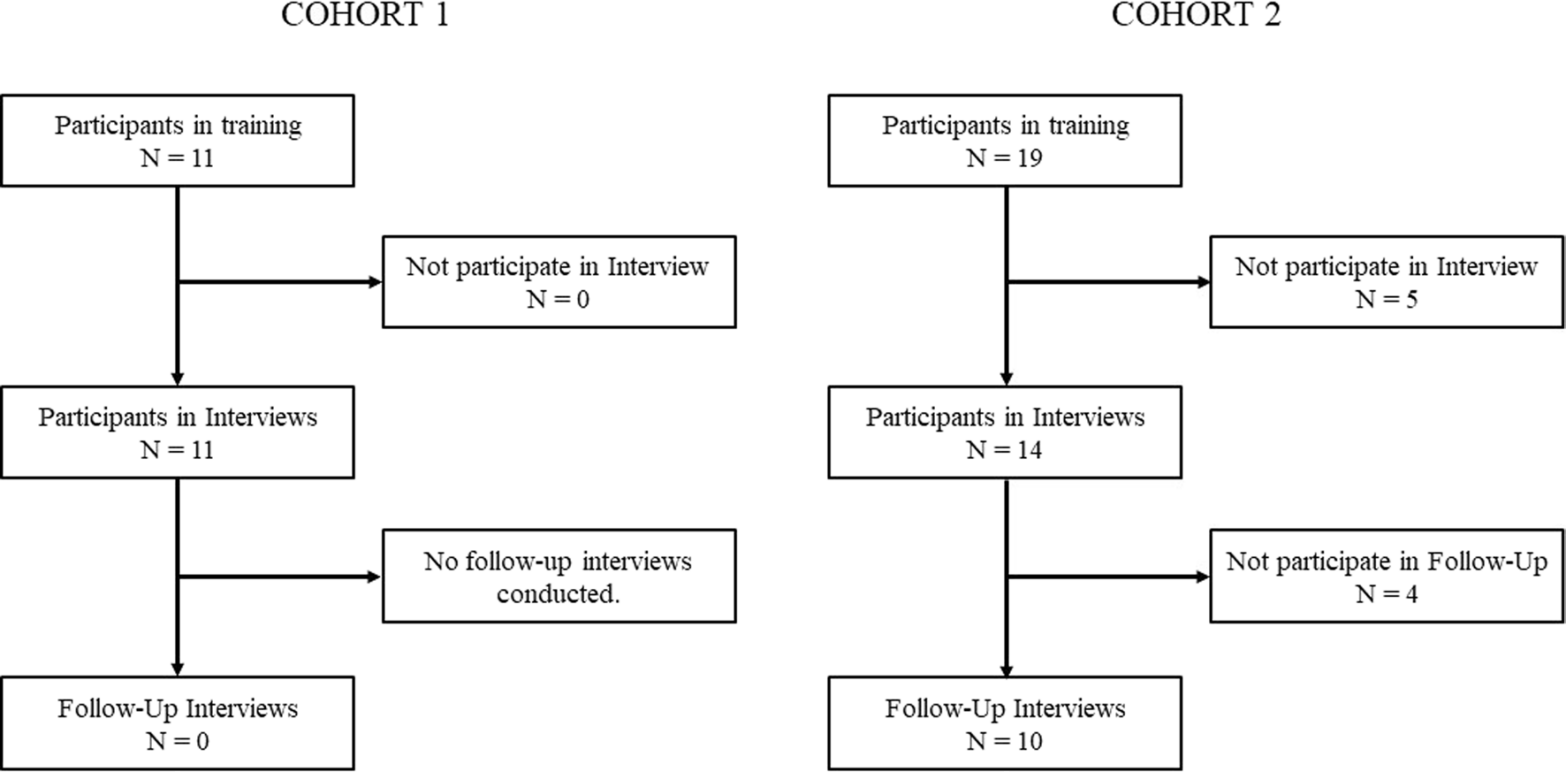
Recruitment

The interviews were conducted from September 2018-July 2020, partly in person and partly by telephone, with OHPs who participated in the clinical training course in basic mental health care. The first training cohort counted 11 OHP-participants, the second training cohort counted 19 OHP-participants. In the first training cohort, interviews were conducted with all 11 participants, solely at the beginning of the training. In the second training cohort, the interviews were conducted at the beginning with 14 of 19 participants of the second training. 10 of those 14 participants were reached for follow-up-interviews at the end of the training, again. All in all, we therefore conducted 35 Interviews with 25 OHPs. The interview guide (own development for this study) focused on: first the OHPs motivation to participate, second, OHPs professional experience with mental health topics, third, the role of company managers towards mental health in their company. In case of interviews conducted at the end of the training, we asked about individual development during the training. For an overview of the interview guide see appendix. For an overview of our recruitment, see Fig. 1.

Participation in the interviews was voluntary; a total of 25 of 30 OHPs took part in the interview, which corresponds to a proportion of 83.3%. Out of the 25 OHPs 6 were male (24%) and 19 were female (76%). Audio recordings of all interviews were made and transcribed. The interviews lasted an average of 26 min, varying in length from 11.00–34.07 min.

### Procedures of analysis

Being interested in a field with yet rather little existing research and thus not having direct hypotheses, we followed a qualitative and hermeneutic approach to our data. The interviews were then analyzed using Qualitative Content Analysis [47], one of the most often used interpretational method of qualitative data [48]. Our analysis was done by using the software MAXQDA [49]. Methodologically, we followed the procedural scheme of the content structuring content analysis. With regard to category creation, we proceeded in a mixed deductive-inductive manner by pre-formulating initial categories based on our familiarity with the topic. We consensually agreed on three initial main categories of analysis, that loosely matched with the structure of our interview guideline. These categories were: 1) the experience of one's own role as an OHP, 2) the organizational culture in respect to mental health issues and 3) the cooperation with other company actors and especially managers. The participants also reported on the experience and symptomatology of their employees, the participants' evaluation of the training and their own level of knowledge and learning gains. In the present study we focus primarily on the results of the points 1)-3). For an overview of the categories of the final analysis see Table 1. The evaluation of the training will be presented elsewhere and is currently still in progress.

**Table 1:** Categories of analysis

1. Experience of one’s own role as an OHP
1.1. Organizational and institutional conditions of the OHP’s work
1.2. Personal tasks as an OHP
1.2.1. Towards patient
1.2.2. Towards the company
2. Organizational culture concerning mental health issues
2.1. Perception of the current situation
2.2. Desired developments of organizational culture
3. Perception of other company actors and especially company managers
3.1. Supporting actors and institutions in the company
3.2. Perception of company managers
3.2.1. Relevance, characteristics, behaviors, situation of managers

Our units of analysis were structured in such a way, according to Kuckartz [47], that each individual interview was treated as a sampling unit. As a context unit we defined closed trains of thought that could also extend over a single contribution (i.e., could also be interrupted by the interviewer). The content unit was defined as being a single word. We first individually analyzed the interviews of first training cohort (N = 11) with the initial category system. Individual analysis means here, that the interviews were distributed on two researchers (TP, MB), of whom each coded one part of the interviews. Conflicts in coding were discussed after fully coding the first 11 interviews and solved consensually, if necessary by consulting a third researcher (ER). On the empirical basis of the 11 interviews, we then collectively and consensually differentiated the initial categories and specified subcategories inductively from the data.

Using the subsequently developed category system we individually analyzed the remaining 24 interviews of the second training. We finally also re-coded the 11 interviews coded at the beginning on the basis of the revised category system. The coding was again done by two coders and again followed by internal discussions for achieving consensus on coding.

### Research team and involvement

Our research was carried out by a team of psychosomatic medicine physicians, psychologists and sociologists. The clinical training course in basic mental health care was offered by the Leadership Personality Centre Ulm (= LPCU) and Ulm University Medical Center, Department of Psychosomatic Medicine and Psychotherapy. The aim of the training was to improve OHPs clinical ability to deal with employees that suffer from psychosomatic or mental ill-health, with focus on the individual level. Some of our authors (ER, HG, MH) worked as trainers in the course. Some interviews were conducted by an interviewer who was known to the participants as a lecturer in the training. Possible effects on our analyses are discussed later in our limitations section. Researcher involvement was reflected in our analysis and the analysis was conducted by researchers (MB, TP, SB) not involved in the training. By this, an independence of the final analysis from the in-field-perceptions of the trainers could be achieved.

## Results

The main categories reported by the OHPs along the given interview guide were: first, the self-perception of the OHPs’ situation; second, a description of the organizational culture towards mental health issues and third, a description of the behavior and attitude of the company’s managers, which was (in the perception of the OHPs) determined by managers qualification and selection.

### Self-perception of the OHPs: highly engaged between patient care and commitment to the company

The participants described themselves as being very engaged in their work and reported a high commitment, despite difficulties in dealing with sometimes conflicting interests in their work.

Many of the OHPs reported to enjoy taking high responsibility in their company and expressed a motivation to develop structures for an adequate handling of their employees. For example, one OHP working in a bigger company reports his high motivation to achieve something for his employees and his company:

OHP: “It’s quite exciting! For me, that was always the point: if you do occupational medicine, then you best do it in a large company, because it's more exciting then (…). I have completely different structures, you know. I can rely on a social service, we have our own health insurance, we have sports programs, we now also have our own occupational health management department, we are also represented in a steering committee as occupational physicians. Of course, you can achieve much more here. (I5)

This engagement and enthusiasm in work is also present in the following quotation:

OHP: "... taking time for (...) employees, and I also find that very positive in the company compared to, well (...), to working as a resident doctor for example, because you simply have the time. And I take my time; because I think, that I can do that quite well: really listening to people, showing empathy, but also leading conversations; of course, not with everyone, that's clear. But I think I can do that quite well and I also think, that I can mediate quite well between managers and employees." (I11)

As a particular challenge in their job, they reported meeting the high demands in an overdetermined environment. Overdetermined means in this context, that other actors’ interests in the company are in conflict with each other and that the physician not only has to prevent sickness and initiate treatment, but at the same time inevitably has to take sides in a diffuse and complex situation.

Interviewer: “So, your role in that context, how would you describe that? What is your main task?”

OHP: “Well, that is what I thought as well (…) that was quite difficult for me, perhaps. The employee wanted me to take part, because we, as I said, have had an occupational rehabilitation talk with him before. And some more talks, when he always expressed towards accusation of mobbing. And then, I realized that today in the talk, too, what role do I have? What can I really do, or what does he expect now? What do the others expect? Should I say something? How far do I distance myself? What else can I do (laughing)? Yes, that was a little difficult for me.” (I12)

The OHPs thus play an important role and participate actively in conflicts between the needs of employees versus the constraints of the company. This becomes very apparent when, for example, rehabilitation conditions are discussed.

OHP: “So the issue “rehabilitation”, how can I accompany that well? How can I prevent burnout? Because many (employees) come to see me with the overburdening, and they explain their symptoms: I have sleep disorder, I have a headache. How do I find a good recommendation, for the employer, too? What cannot be considered? How much do I respond to the wishes of the employee, because sometimes it gets quite absurd: «I have to work in home office five days and I only can have minitime-tasks.»” (I24)

Among these more general conflicts, especially the treatment of mental disorders was described as a major task by the OHPs and caused a lot of insecurity in them:

OHP: “I noticed that I somehow lack the tools to handle mental, psychosomatic or psychiatric disorders, which appear again and again in work life. And there are situations in conversations: I’m partly not trained for, not prepared for, and I have to solve them somehow with my own common sense. And there were situations, again and again, which were really difficult and where I wished for some support. (…) And this is where I recognized, that I’m partly not proficient in and in part I’m not able to conduct those conversations, too.” (I15)

At the same time, the OHPs repeatedly report that they feel their patients trusting them and speaking openly about their issues. This trust by the employees however causes an even bigger insecurity of the OHPs.

OHP: “But I felt the boundary for me. I don’t know how to help him further. Trust was there, yes, so that you had the feeling, he wants to talk more deeper about the issues, but I didn’t know, how should I do that with him.” (I8)

Despite their high motivation, the OHPs feel restricted by their knowledge and see the need for further education.

OHP: “But really rather in the acute situation, someone comes with a complete unknown issue, gets an appointment and then unburdens himself so to speak, his troubles or cries than all of a sudden in front of me, because he is just, or she is just completely overstrained. So, handling that, having conversations techniques and so forth, these are things I lacked in certainty, a little bit in the past.” (I5)

After completing the training, the OHPs reported being more certain on the subject of mental health and more capable on how to treat employees with psychical strains or even suffering from mental illness. In general, they conclude that the training helped them on a practical level concerning everyday work-problems, too.

OHP: “Very appropriate and very necessary (both are laughing). Well, because it is helpful now with the daily work, too, to classify things and even conversations, you become a little more certain in conversation techniques and you dare to say: Yes, come again later. Like some sort of psychological surgery. Well, I don’t want to overstate that, but just to say: Yes, we can talk again about that and, and after all, well, that has helped much.”

Interviewer: Do you have the feeling that something changed a little bit over the time you were here? For example, your motivation or how you handle such conversations?

OHP: “Yes, you simply become more confident, a little bit, and you simply dare to do that, so to say. Yes, for example I’ve got a lady with eating disorder, I consequently summon her every four weeks. She is ill for a very long time, too, is doing her rehabilitation, and simply to stabilize her a little bit, I tell her: Just come, we can talk. Although I always say I’m not a therapist in that sense, but we can talk it over.” (I2)

### Perception of organizational culture

The OHPs also described the prevailing organizational culture, mainly towards mental health issues. One group of OHPs reported a relatively open culture:

OHP: "So I think this is taken very seriously and at (my company) there is not only the medical service, where I am working now, but there is also the internal counseling service, which is more or less a psychological counseling center, where all employees can turn to and (...) that shows that the topic is taken seriously and that they also try to give assistance somehow or to provide the employees with counseling, so to speak, and so I think this is taken seriously.” (I18)

In line with that, some OHPs gave descriptions of elaborated psychological and psychosocial institutions (e.g., psychosocial counselling) in their company and reported, that issues of mental health were taken serious on almost all levels of their company. The openness within the company towards mental health issues was also reported in comparison to other companies.

OHP: „It becomes apparent, I think, that we really take up the issue psychical risk assessment in the company. I think one can always do more, but that it was integrated properly… I know that from other companies, where it’s not implemented, so I think that we are quite ahead in our company. For example, that (we) offer a compulsive training on risk assessment and psychical health. A bargaining agreement was properly singed, to both topics and management as well as workers’ council pushed these topics. We also not only have physicians, but have social workers in the company, too, and we cooperate well. All of that are signals. And that the training for managers was offered, compulsive on the issue and offers were established, too, for example a contact to and rapid integration in a psychosomatic clinic and coaching, training for resilience building and moderation in conflict-talk and such things.” (I5)

Other OHP reported more skeptically that mental health-topics were treated rather careless or with little attention in their company:

OHP: "…nice words and nice pictures, but whether it is really what is wanted, if, in other words, we really want to reintegrate everyone, I don't know. It's also a question of, well, the work has to be done and we're not... we can't keep all the chronically ill here. That is also an announcement.” (I19)

While these reflections describe at least a certain interest in the topic from the company side, mental health seems to be met with open rejection in other companies. Here, the interviewees describe that the topic is well known in the company, but is actively resisted.

OHP: "the management thinks very little of the topic of psyche" (I6)

Most of the time, physicians mainly describe the current situation and do not go into further detail about its background. When this happens, reference is usually made to the cost-effectiveness of the measures and the company's fear of putting them at risk by addressing psychological issues too much.

OHP: "...because the company of course also sees the days of incapacity to work continue to rise. Of course, we (the physicians) want to do something against that, but if you then say: “we would recommend this and that”, or then they (the company) think “Oh no, that, somehow, is too much of an effort or is expensive or could somehow, yes, bring negative results." (I6)

Here a situation is described where companies are aware of the strain on employees, but do not want to draw consequences, and are rather afraid that this could have negative consequences for the company (presumably primarily in economic terms). After all, there are also physicians who describe an open ambivalence in their company, in the sense of a contrast between set out concepts and everyday practice, which falls far short of these ideals:

OHP: "…it really depends on the people who have to do with it, …on the responsible person in the personnel department, on the manager, it really depends on them.” (I32)

### Perception of company managers

A common claim in our interviews was, that much of the realization of a company's culture depends on the managers. According to that argument, managers play a crucial role for the mental health of the employees.

OHP: “I’m doing this now for seven years and I’m here in this company for seven years. And leadership is the crucial factor, which lets the employee enjoy going to work. And once someone enjoys doing something, he learns easily, he works easily, we all know that. And as soon as someone enjoys what he does, he becomes less ill.”

Interviewer: “And you are saying, leadership plays a crucial part – in which way?

OHP: “Completely. So – you cannot differentiate that. If you have a reasonable style of leadership, they (the employees) will come motivated to work.” (I14)

Other OHPs directly confirm this assessment and draw a direct link to employees (mental) health:

OHP: "Well, that's what I actually believe, that a person who is well led and has trust in managers, stays significantly healthier and performs better even in stressful situations and can achieve more and that, of course, negative leadership is often also the cause of mental illness and that's what I experience in many staff rounds." (I24)

None of the interviewees reported that managers and their behavior were unimportant to the mental well-being of employees, even though it was also reported that mental stress is also brought along from home or can arise as a result of interactions with colleagues from the same level of hierarchy.

Also, when asked for what reasons the physicians were consulted by employees, difficulties with managers were named (more than conflict with colleagues and stress form private environment) as an outstandingly important point.

OHP: "I think that plays a very big role... so whether the employees are doing well or not, depends to a large extent on the manager. That's what we see time and again." (I25)

Apart from that, the OHPs also regularly mention that managers are to a high degree a transmission medium of the organizational culture and may embody it in the employees' everyday experience. The otherwise abstract organizational culture manifests itself by this way to the employees in daily work routines.

OHP: "In that case, I don't think that it’s actually a climate that promotes good health. And even if there’s a written statement like 'We have a great health management system and a great psychosocial counseling center': if I then have managers who are known to be incapable of managing employees and leave these managers in their position, perhaps simply transfer them somewhere else and say 'Well, then they should just destroy the employees there,' then I don't know if this is the right way to deal with things. You can sense a certain frustration in me (laughs). Because that's just something that I notice again and again in everyday life." (I16)

Many OHPs criticize the way managers deal with their employees. They claim that most managers are mainly competent in professional, instead of leadership or social skills.

OHP: "That is the problem, that they are allowed to lead without having learned it. Not everywhere, but often. Someone is given a management task who has never proven that he is qualified for it, except by his professional skills. And perhaps you have to look more carefully when someone takes on a management task. Just because someone seems like a “jack-of-all-trades” and can do some great things…, you still somehow have to look if he can also lead." (I26)

From the perspective of the OHPs this is particularly disadvantageous because managers play an important role in the company in three different ways. First their work is largely responsible for how healthy a department is. Second, they realize the fundamental ideas of an organizational culture. Third, they could also fulfill an important role referring ill employees.

OHP: "The manager can notice in time if something is not right with the employee and then he or she can intervene in time, speak to the employee and can ensure that the employee has enough trust and is perhaps sent on to the appropriate help centers or comes to us for advice.” (I19)

All in all, the OHPs’ evaluation of managers remains very mixed for all three areas mentioned. Although some open-minded managers are described, they rarely live up to their responsibility for their employees. The perception of the managers remains rather not empathetic. Sometimes the OHP sense the managers’ own difficulties, such as sandwich positions, but nonetheless for the most part, the OHPs’ descriptions lead to the impression of a rather difficult relationship to the managers.

### Skills of managers: professional versus interpersonal. “Leadership cannot be learned!”

Many OHPs state that a major problem in the collaboration with managers is that managers are usually not selected on the basis of their social, but of their professional skills.

OHP: "For me, it starts at the point in hiring, when management personnel are hired. That leadership quality, in other words, what I call emotional intelligence, is given such a low priority by the leaders compared to supposedly technical competencies, that that doesn't get any real importance!" (I16)

According to the OHPs social skills of managers are of bigger importance for employees than just technological skills or economic expertise. The lack of these skills is identified as a major obstacle to a healthy work environment.

OHP: "Well, I think the problem is that professionalism is so much in the foreground in the selection of managers that social competence usually only plays a subordinate role in their evaluation. This is why executives get into managerial positions even though they are not really suitable for it and I personally think that the (...) professional qualification is easier to develop than developing the (...) personality structure in the person himself, so that he is a good manager.” (I29)

One possibility to deal with this issue are educative workshops. Here however, the OHPs often report that such workshops are unfortunately usually only attended by managers who are already competent in dealing with their employees. In contrast, managers whose management style would lead to stress with the employees would reject such offers and could not be "forced" to be interested in the topic.

OHP: "The problem I see is that the people who have already reached the point where they say "Yes, I have my own role as a manager, i have a responsibility for my employees and I have to see how I deal with them”. They gratefully accept this and improve their skills, and they are very open to it. But the managers who have no awareness of the problem, who say "I'm not to blame for the situation, it's all the employee's fault and I'm great and whatever", you can't get to them. You can train them as much as you want, they don't come to any training voluntarily (now and then to a compulsory training course to which they are sent), and those are the ones you simply can't reach at all and who nevertheless ruin everyone.” (I29)

## Discussion

This study investigates OHPs attitudes towards mental health care at the workplace and the perception of their own role as well as contributing factors in this field such as the impact of organizational culture and the importance of leadership. Qualitative content analysis was based on semi-structured interviews with OHPs interested in this topic.

The OHPs in our sample were highly motivated to learn more about supporting their mentally ill employees in general and wanted to become more capable and self-confident in dealing with psychical and psychosomatic disorders. At the same time, many of the OHPs reported that their initial knowledge of psychosomatic medicine and psychotherapy was subjectively insufficient and that they often felt overwhelmed working with such patients. Concerning the organization in which they were working (with focus on the organizational culture and leadership practice) they reported support as well as rejection, which the OHPs perceive as major influence, as both promoting and hindering factors for their work with employees suffering from mental illness.

### Key findings

Many OHPs stated that their role was characterized by first, the will to support their employees and second, the (sometimes conflicting) need to fulfill the demands of the company or leading management. In positioning themselves in this tensioned field, many OHPs showed a strong sense of alliance, connectedness and trust with their employees and rather distanced themselves from the interests and demands of their company. Our findings here support older research on the field of OHPs values [50]. The general descriptions of conflict between employees and company’s interests go along with theoretical concepts, that describe the work of an OHP as a management of (sometimes ethical) conflicts [40] and suggests that many OHPs rather ally with their employees than with their company. In line with this many of our OHPs strongly orientated to a therapeutic model of interaction [29] with their employees, seeking the level of trust prevailing in the relation between a general practitioner and a patient.

Furthermore, the OHPs gave detailed descriptions of their working conditions. They reported that their work was highly dependent on environmental variables. One point repeatedly mentioned was that the organizational culture towards mental illnesses in general and its transformation from abstract company guidelines to daily routines by means of the managers was of great importance. This falls in line with existing research showing a link between different types of organizational culture and mental well-being of employees [22, 24]. The OHPs often stated that a company’s attitude towards mental health lays the ground for the well-being of their employees and affects their own working conditions and possibilities to support mentally ill employees. Without directly referring to it, our OHPs confirmed conceptual connections as formulated in the research on psychosocial safety climate [33].

Concerning the organizational culture, many OHPs observed a change from a dismissive to a more open attitude of companies towards mental health at the workplace. This was reflected in descriptions of written company-guidelines, brochures or intranet-pages. In addition, OHPs also reported about the existence of programs for employees and managers suffering from mental illness. Overall, the OHPs mentioned an officially more open attitude in general, which is often not further described. These descriptions were sometimes combined with reports about existing psychosocial support-service centers or individuals working in management or the human-resources-department. These actors were described as generally open-minded towards mental health, which again were reported as proof for the general more open attitude of the company. In the light of Schein’s theory of organizational culture [20] as outlined in the introduction, culture is observed by the OHPs primarily on the first and second level, where first written company-guidelines or brochures make up *artefacts* and second the reports about the talk and behavior of e.g., top-management about mental health issues can be understood as *espoused beliefs and values*. As expected in an interview of limited scope, the OHPs did not explicitly describe their company’s *basic underlying assumptions.* Nevertheless, at this point the OHPs hint at a problem in companies, where change in respect in to a more open attitude towards mental health has not yet occurred in depth and that an in-depth transformation seems to be necessary to support employee’s mental health in the long run.

Concerning the organizations attitude towards mental health a remarkable gap seems to exist: while many OHPs stated the presence of a more open attitude towards mental health on an outer level of culture (e.g., in the form of guidelines and so on), many (often the same) OHPs criticize that these artifacts have not yet led to a deeper change of the organizational culture on the level of espoused beliefs and basic assumptions. This only superficially open-minded organizational culture then leads to more problems in supporting mentally ill employees directly (via initiation of treatment) or indirectly (via influencing working conditions). And even concerning Schein's level of artifacts and behaviors, many OHPs also reported a situation in which the respective topics of mental health at the workplace ranked second to economic issues and were either ignored or openly rejected. Here the OHPs describe a situation in which the evidence that a high orientation on psychosocial factors supports economic productivity [51] has not been implemented sufficiently in their companies. Only in the best case was a positive development reflected in established institutions such as psychosocial counselling in the company. In many others cases physicians were more sceptic even concerning an outer level of culture.

In line with existing research on the connection between leadership and employee well-being [25–28] the cooperation with the company’s managers was often described as a major influence for the physicians and the employee’s situation. While the organizational culture of a company remained a rather elusive influence, the direct behavior or attitude of leaders was almost consistently described as being a major and very concrete influence for their own and their employees’ working conditions. This differential influence of culture and (middle-) management falls in line with theories on organizational culture and leadership that suppose, that organizational culture is primarily connected to concepts of management by attitude, priorities and decisions of the upper management, CEOs or company founders [21], making delayed effects of a changing organizational culture to the level of a middle management possible. Despite an obviously slowly changing organizational culture, working with difficult managers was still a major source for stress at work for most employees.

Not surprisingly such constellations were described as major obstacles in the work of the OHPs in the way that many of the mental illnesses they had to deal with were a result of the stress employees develop because of the relationship with their managers. Second to that, the OHPs also reported that they often had to work or negotiate directly with managers. Here they painted a critical picture and described that the cooperation with managers of the company was mostly difficult, because managers didn’t seem to have the necessary interpersonal skills to deal sufficiently with their employees. In contrast to existing research in the field of training on leaders’ behavior [52], many OHPs were rather skeptical about the possibilities to teach leaders the necessary skills. Contrarily many OHPs made the observation that such skills *“could not be learned”* afterwards and demanded a pre-selection and hiring of managers based more on social instead of professional or technical skills. Even though some physicians showed empathy for the often-difficult situation of managers, the willingness to really take over the perspective of managers and to perceive the collaboration as overall collaborative was rather low.

A healthy relationship between employees and managers as part of a psychosocially safe climate, is—in the long run—of importance for a company’s productivity. Our results therefore go in line with the ideas of psychosocial safety climate [12, 13, 35, 53] and emphasize the importance of the managers’ relevance for stress at work. Therefore, as Dollard et al. [53] argue, it is necessary not only to focus on individual coping skills, which one (for example: a physician) can give burdened employees as advice and let them solve their problems individually, but to consider the organizational context of stress, such as top-management decisions and managers behavior towards mental health issues.

### Strengths and limitations

Our study gave insight into the subjective view of OHPs dealing with mental illnesses. In our study we analyzed subjective reports about OHPs’ work with employees at risk of or suffering from psychosomatic or mental illness. By doing this, we gained profound insight into the OHP’s subjective perception of their environment. This approach also limits the scope of our study, since we did not collect data on objective properties of the OHP’s companies or working conditions. This in turn might allow further understanding of the interplay of such external variables and the subjective experience of an OHP. For our analyses no data was available about the size of the companies, as well as their sector.

Our sample consisted of highly motivated OHPs interested in learning more about mental health care. This might lead to a biased perception compared to the overall population, which might have a smaller awareness of the issues. On the other hand, the strength of this approach is exactly this selection: Only OHPs who are committed to mental health issues will recognize what promotes and what hinders improvement in the organization.

Furthermore, the interviews were in part conducted by trainers of the training. This could cause a certain social desirability bias among the participants, as well as a bias among the interviewers for certain aspects of the training and work of the OHPs. We dealt with this in the process of research in the way, that researchers with direct contact to our participant during the training did not directly analyze our interviews but only worked with the final analysis in summarized form. Finally, due to personal and economic reasons, it was not possible to have all interviews being fully coded by two coders independently. This might have augmented the reliability of our results further, even though only little divergences between the coders occurred and the results proved robust independent of which coder found them.

## Conclusions

Our results have shown that the work of an OHP in helping employees suffering from mental illness is highly determined by context variables and cannot be isolated to a one-dimensional model of prevention or treatment. On the one hand, this underscores the importance of improving the OHPs’ competences in dealing with employees (as well as managers) suffering from mental illness. On the other hand, this also means that future interventions for OHPs should foster awareness of the importance of the organizations (psychosocial) safety climate and its impact on employees (mental) health. OHPs should ideally be able to keep in mind the overall picture of the complex construct of mental health at work. This overall picture should include the psychosocial impact of working conditions and interpersonal relations at work, as well as considering the general attitude of the company towards mental health issues, which is expressed through guidelines on the topic and top-management decisions. OHPs need to consider the company’s net of relationships and hierarchies, since they are actors between different levels in the company. They have to be able to position themselves according to the case they are dealing with and balance the needs of employees and the interests of the company, especially when their patients suffer from psychosocial strains and mental illness. All this leads to the point that future interventions to support OHPs should carefully address these dimensions and encourage them to position themselves confident towards other actors in their company. In addition, pointing out the ethical dimensions of their work could further help OHPs in choosing their actions more consciously and self-determined. Future interventions for OHPs should therefore and furthermore clearly address the systemic dimension of the work of an OHP.

Considering all these factors, which form of intervention would be best to support OHPs in helping employees suffering from mental illness? In our training the insecurities of the OHPs in dealing with mental health at the workplace could be—at least in part—successfully addressed. Even though many OHPs came to a mixed evaluation of their working environment, many perceived more possibilities to support employees more adequately after participating in the training. Many felt more secure in managing conflicting interests in their work and dared to position themselves more confident in the company. Therefore, it could be recommended to provide such trainings and workshops for a wider audience of OHPs. Another tool to support OHPs could be to encourage exchange between OHPs of different companies, for example in the form of an open group setting. During the training, many OHPs reported that interpersonal exchange was of high importance to them and stated that they wanted to keep in touch to hold onto that form of an open group setting.

One more possibility would be to grant OHPs a advisory function. It seems conceivable for example, that OHPs could support company management e.g. in dealing with psychosocial conflicts on different levels of the organization. The prerequisite for this would be sufficient familiarity with the significance and implications of the topic, which could be conveyed through appropriate courses.

In the same way and due to the high healthcare and economic impact of common mental illnesses, it would furthermore seem reasonable to expand training courses on stress-associated somatic as well as mental health aspects in occupational medicine, e.g., as part of a comprehensive occupational health management program.

## Appendix: Interview guide

**Topic 1: Motivation for participation in the training and expectations**

- Main question:
  - What were your reasons for participating in the training?
- Optional in-depth questions:
  - How reasonable and necessary do you consider the training for yourself?
  - Did your original motivation for participating change during the course?

**Topic 2: Own workplace and occupational experience**

- Main question:
  - Which issues of mental health play a role for an occupational health physician in a company?
- Optional in-depth questions:
  - With which issues do employees seek your help?
  - How does your company treat mental health issues?
  - Who is important multiplier for these issues in your company?

**Topic 3: Role of leadership regarding mental health issues**

- Main question:
  - Which role does leadership play for mental health issues in the company?
- Optional in-depth questions:
  - Which role could you play?
  - What hinders you from taking this role?
  - How could managers be supported?

**Topic 4: Personal development and progress**

- Main question:
  - Did something change in your knowledge, skills and attitude of specific psychotherapeutic topics?
- Optional in-depth questions:
  - What are your strengths and weaknesses in learning about psychotherapy?
  - Did something change or improve in your personal life and relationships, too?
  - Is there anything you would like to talk about, that we did not address?

## Data Availability

The datasets generated and/or analyzed during the current study are not publicly available due to anonymity of participants but are available from the corresponding author on reasonable request.

## Abbreviations

OHP: Occupational health physician
LPCU: Leadership Personality Center Ulm
